# Supplementary material for: ATP synthase hexamer assemblies shape cristae of Toxoplasma mitochondria
Source: Nat Commun. 2021 Jan 5;12:120. doi: 10.1038/s41467-020-20381-z (PMC7785744; doi:10.1038/s41467-020-20381-z)
Supplement: Supplementary file 1 — Supplementary Information [file 41467_2020_20381_MOESM1_ESM.pdf]

Supplementary Information

ATP synthase hexamer assemblies shape cristae of *Toxoplasma* mitochondria

Alexander Mühleip[1,2†], Rasmus Kock Flygaard[1,2†], Jana Ovciarikova[3], Alice Lacombe[3], Paula Fernandes[1,2,3], Lilach Sheiner[3*], Alexey Amunts[1,2*]

[1] Science for Life Laboratory, Department of Biochemistry and Biophysics, Stockholm University, 17165 Solna, Sweden.
[2] Department of Medical Biochemistry and Biophysics, Karolinska Institute, 17177 Stockholm, Sweden.
[3] Wellcome Centre for Integrative Parasitology, University of Glasgow, UK.

† These authors contributed equally to this work.

* email: lilach.sheiner@glasgow.ac.uk, amunts@scilifelab.se

This file includes:
Supplementary Figures 1 to 15
Supplementary Tables 1 to 3

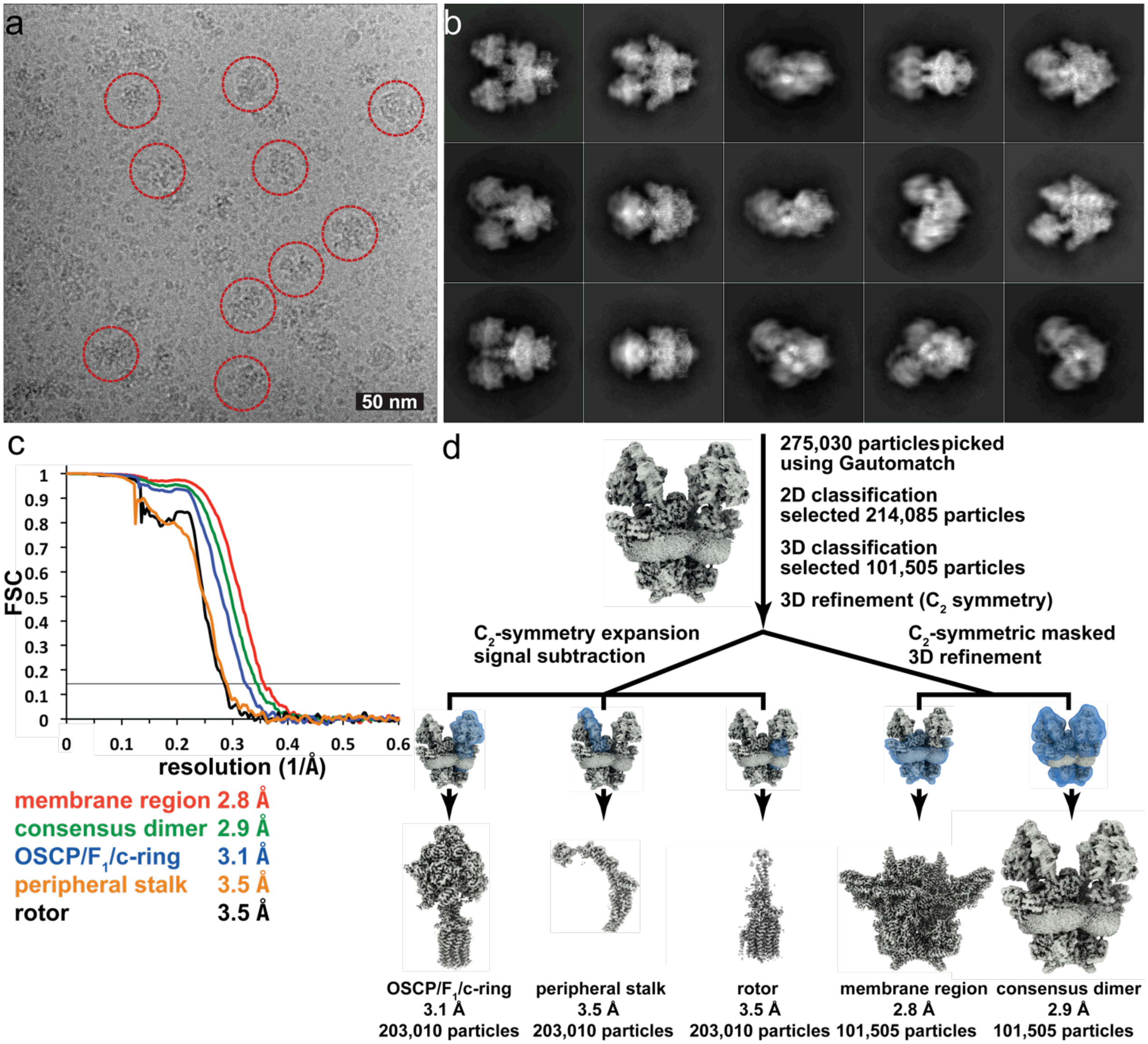

Supplementary Fig. 1 | Cryo-EM data processing of the *T. gondii* ATP synthase dimer.
a Representative micrograph. **b** 2D class averages. **c** Fourier Shell Correlation (FSC) curves showing the estimated resolutions of ATP synthase dimer maps. **d** Data processing scheme resulting in five final maps.

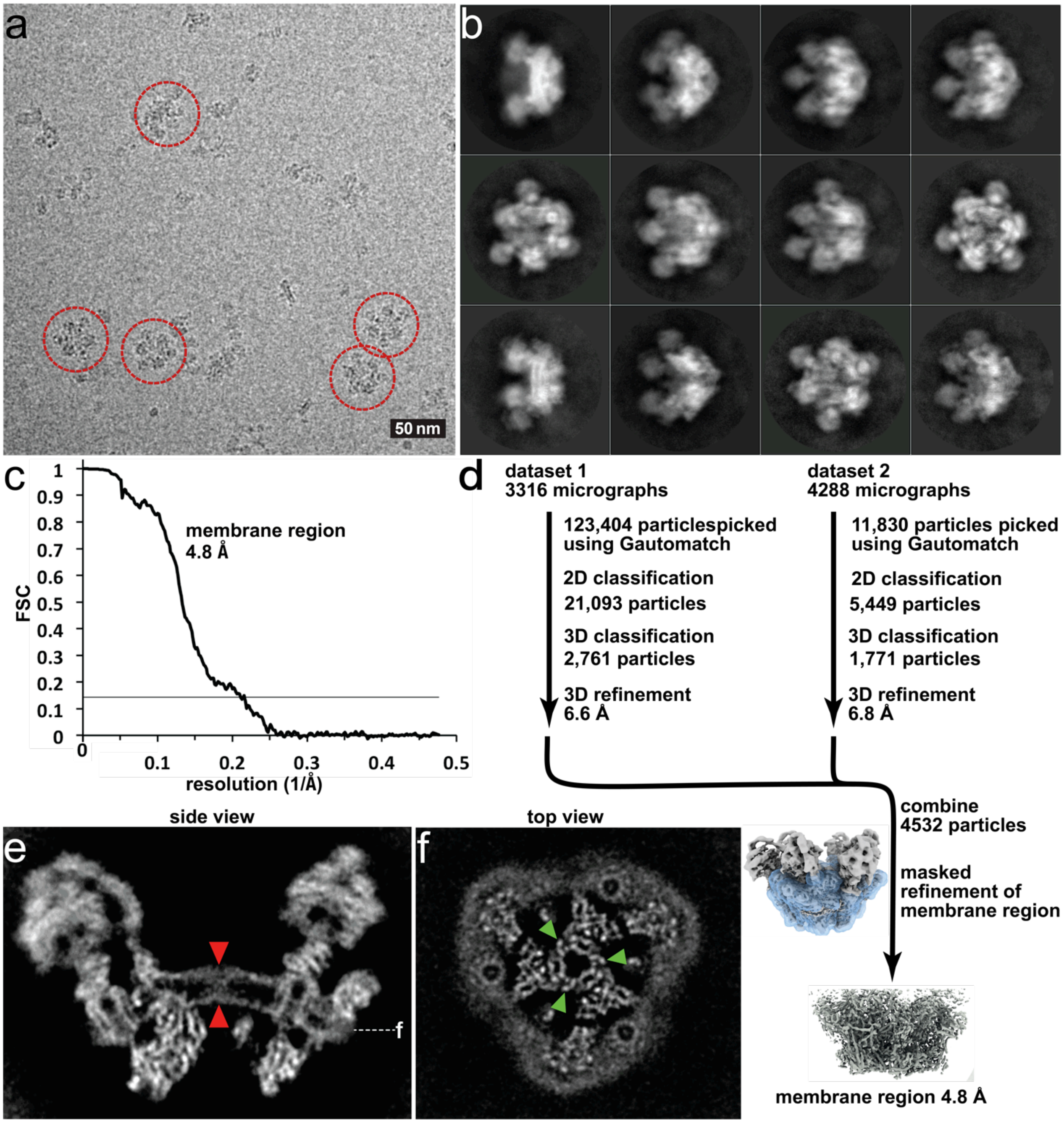

Supplementary Fig. 2 | Cryo-EM data processing of the ATP synthase hexamer.
a Representative micrograph. **b** 2D class averages. **c** Fourier Shell Correlation (FSC) curve showing the estimated resolutions of ATP synthase hexamer map. **d** Data processing scheme resulting in one final hexamer map. **e** Slice through the hexamer map (side view), central membrane bilayer is indicated. **f** Slice through the hexamer map (top view), ATPTG9 copies are indicated.

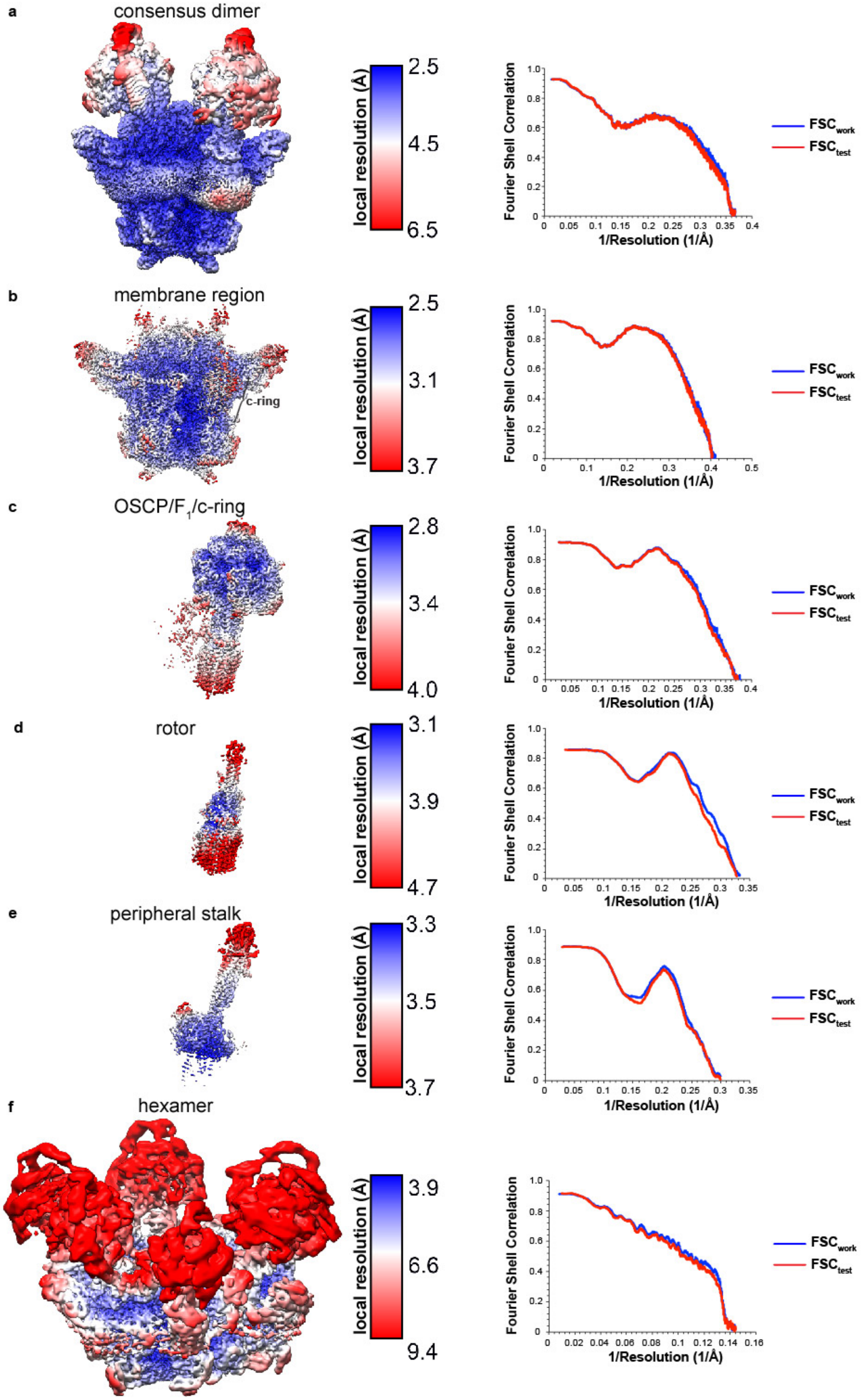

Supplementary Figure 3 | Local resolution estimation and model validation.

Left column shows cryo-EM maps colored according to local resolution as estimated by RELION. Right column shows Fourier shell correlations FSC_{work} and FSC_{test} for validation of models built and refined into the respective maps. **a** Consensus dimer. **b** Membrane region. **c** OSCP/F_1/c-ring. **d** Rotor. **e** Peripheral stalk. **f** Hexamer.

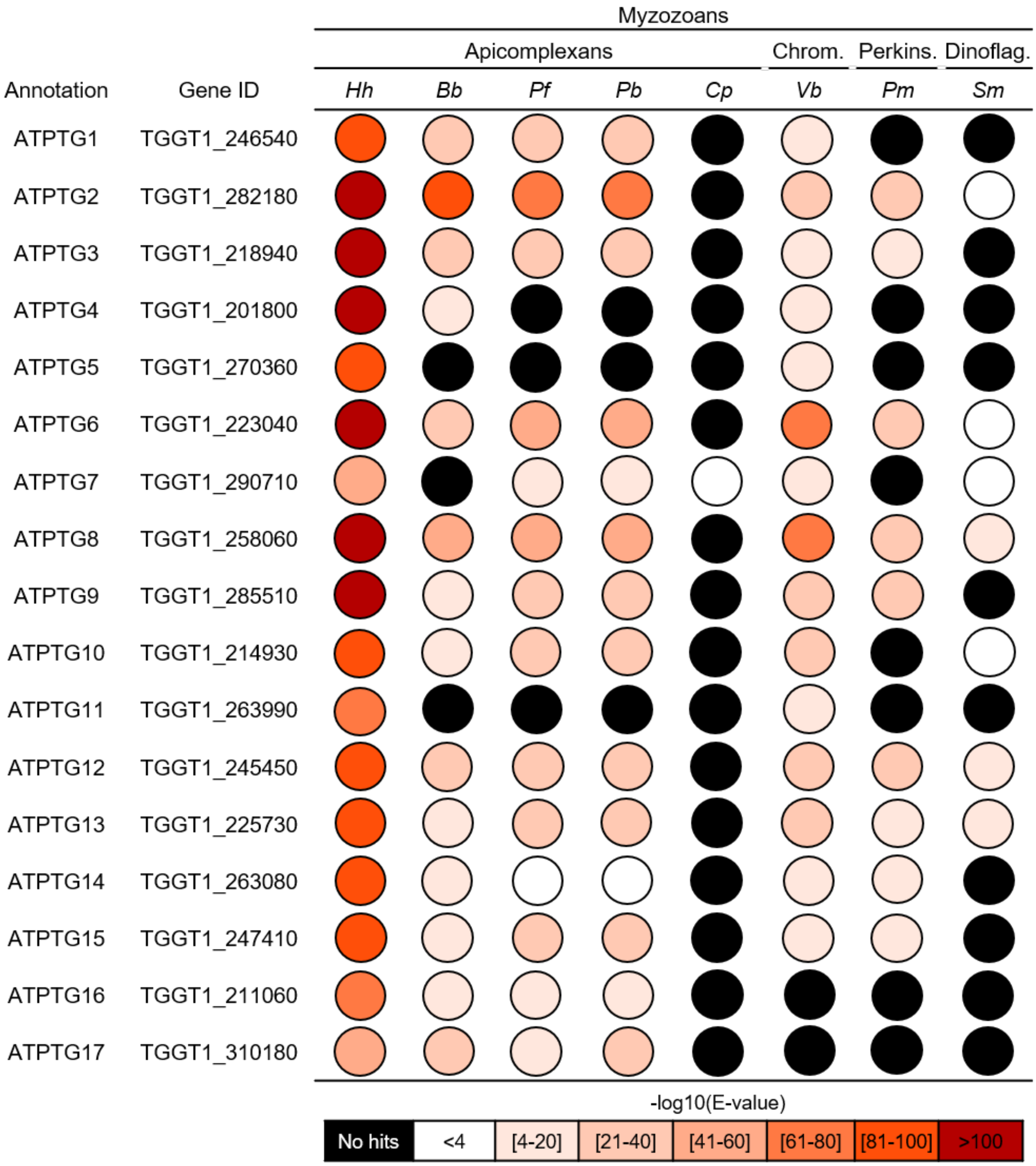

Supplementary Fig. 4 | Conservation of *T. gondii* ATP synthase subunits in Myzozoa. Identified subunits are coloured according to similarity score, represented as -log10 (E-value). Chrom., Chromerida; Perkins., Perkinsozoa; Dinoflag., Dinoflagellata. *Hh*: *Hammondia hammondi*; *Bb: Babesia bovis; Pf: Plasmodium falciparum;* *Pb*: *Plasmodium berghei*; *Cp*: *Cryptosporidium parvum*; *Vb*: *Vitrella brassicaformis*; *Pm*: *Perkinsus marinus*; *Sm*: *Symbiodinium microadriaticum*. All subunits are found in at least one apicomplexan species, but none is confidently identified in *C. parvum* (an apicomplexan possessing mitosomes). Additional identified Sarcocystidae homologs not shown for clarity; see Supplementary Fig. 10.

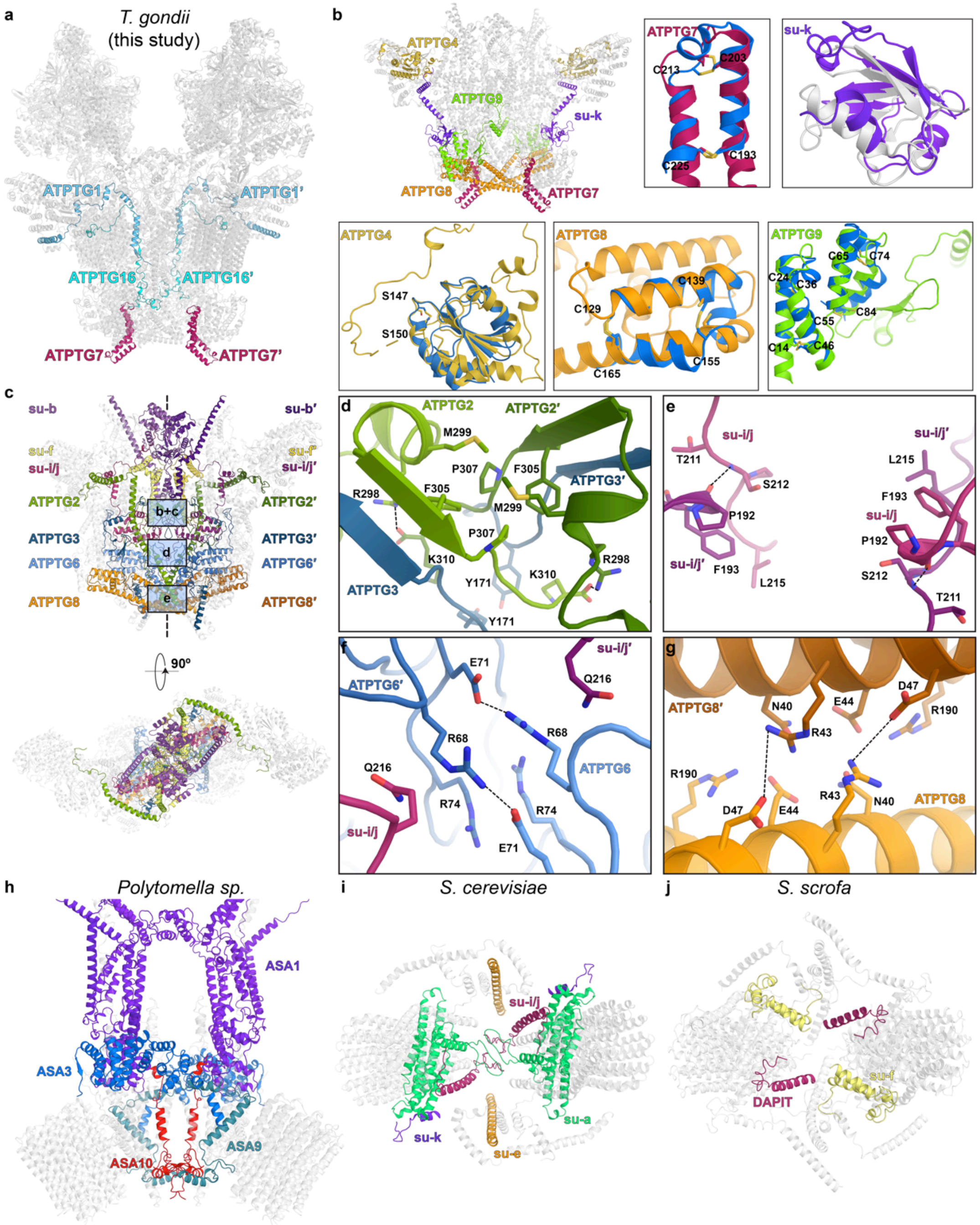

Supplementary Fig. 5 | Subunits and interactions at the dimer interface. a Subunits newly identified from the cryo-EM map. **b** F_o subunits adopting compact folds are coloured. Close-up views show the C-terminal region of subunit-*k* adopting a ubiquitin-like fold (grey overlay, PDB 5X3P)[http://doi.org/10.2210/pdb5X3P/pdb] on the luminal side, ATPTG4 adopts a thioredoxin-like fold in the wing region (blue overlay, PDB 2TRX) [http://doi.org/10.2210/pdb2TRX/pdb] and ATPTG7-9 contain CHCHDs, characterised by two CX_9C motifs (blue overlays, PDB 2LQL)[http://doi.org/10.2210/pdb2LQL/pdb], some of which form disulfide bonds. **c** F_o subcomplex with subunits participating in the dimer interface coloured. Interfaces along the C_2 symmetry axis are indicated. **d-g** Close-up view of dimer interface interactions between ATPTG2 and ATPTG3 **(d)**, subunit-*i/j* (**e**), ATPTG6 (**f**), ATPTG8 (**g**). **h-j** Subunits involved in dimer interface interactions in *Polytomella sp.* (PDB 6RD4) [http://doi.org/10.2210/pdb6RD4/pdb][1], *S. cerevisiae* (PDB 6B8H) [http://doi.org/10.2210/pdb6B8H/pdb][2], and *S. scrofa* (PDB 6J5K) [http://doi.org/10.2210/pdb6J5K/pdb][3].

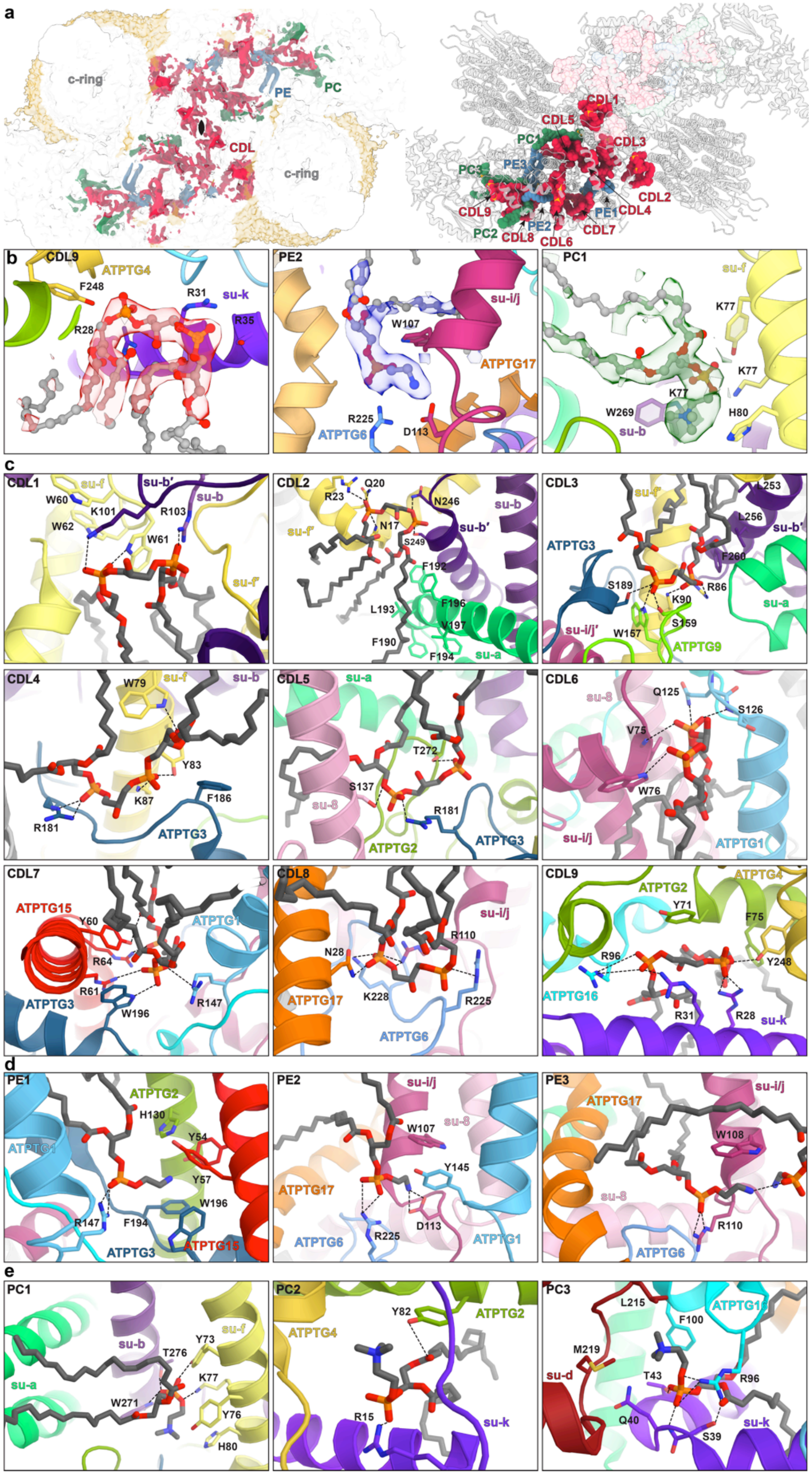

Supplementary Fig. 6 | Native lipids are bound throughout the extended F_o region.
a Top view of the membrane region cryo-EM map (left) and model (right) with bound lipids. The dimeric F_o region includes 18 cardiolipins (red), 6 phosphoethanolamines (blue) and 6 phosphocholines (green). **b** Bound lipids of each the three identified types shown with cryo-EM map density for the respective ligand. **c-e** Lipid binding sites of the cardiolipins **(c),** phosphoethanolamines **(d),** and phosphocholine **(e);** interactions with distances <4 Å are indicated.

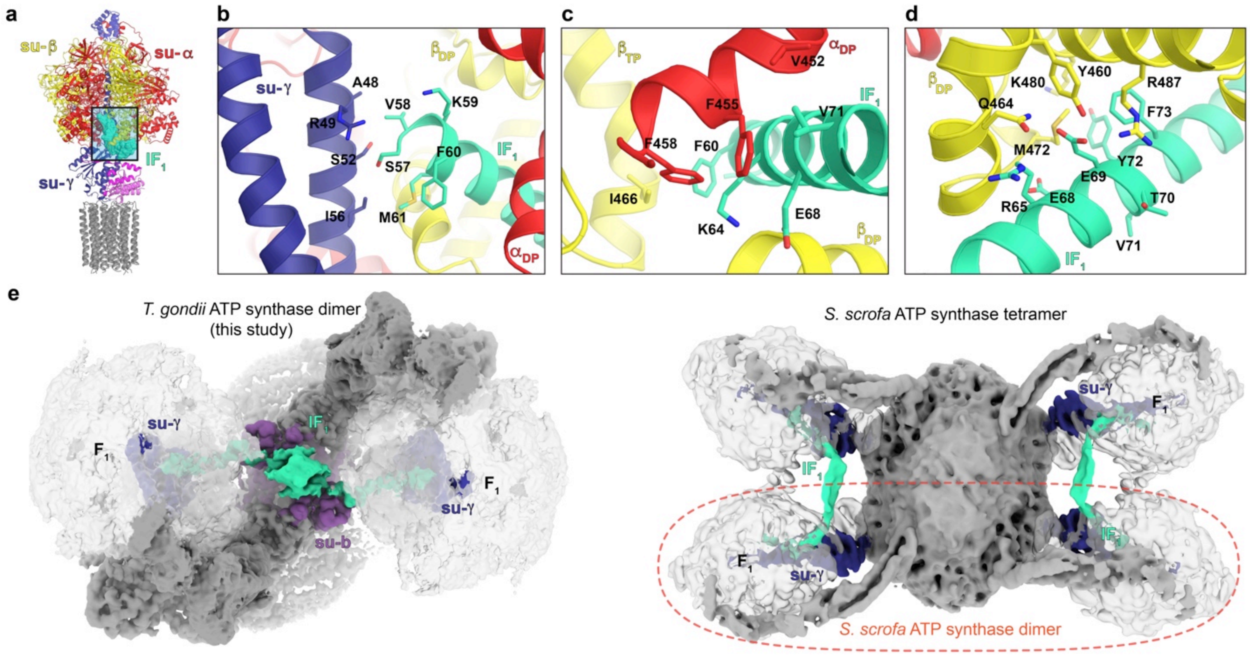

Supplementary Fig. 7 | *T.gondii* ATP synthase dimer contains intradimeric IF_1.

a Model of the OSCP/F_1/c-ring with bound IF_1 (dashed box). **b** IF_1 α-helix extending towards the central stalk subunit-γ in the center of F_1. **c** IF_1 helix inserted into the $(\alpha/\beta)_{DP}$ interface. **d** IF_1 interactions with subunit-β_{DP}. **e** Cryo-EM maps of the *T. gondii* ATP synthase dimer (current work), where IF_1 binds in the intra-dimeric mode and the *S. scrofa* ATP synthase tetramer (EMD-0667) [https://www.ebi.ac.uk/pdbe/entry/emdb/EMD-0667], where IF_1 binds in the inter-dimeric mode, and F_1 (transparent grey), F_o (dark grey), IF_1 dimer (cyan), subunit-γ (dark blue) are highlighted.

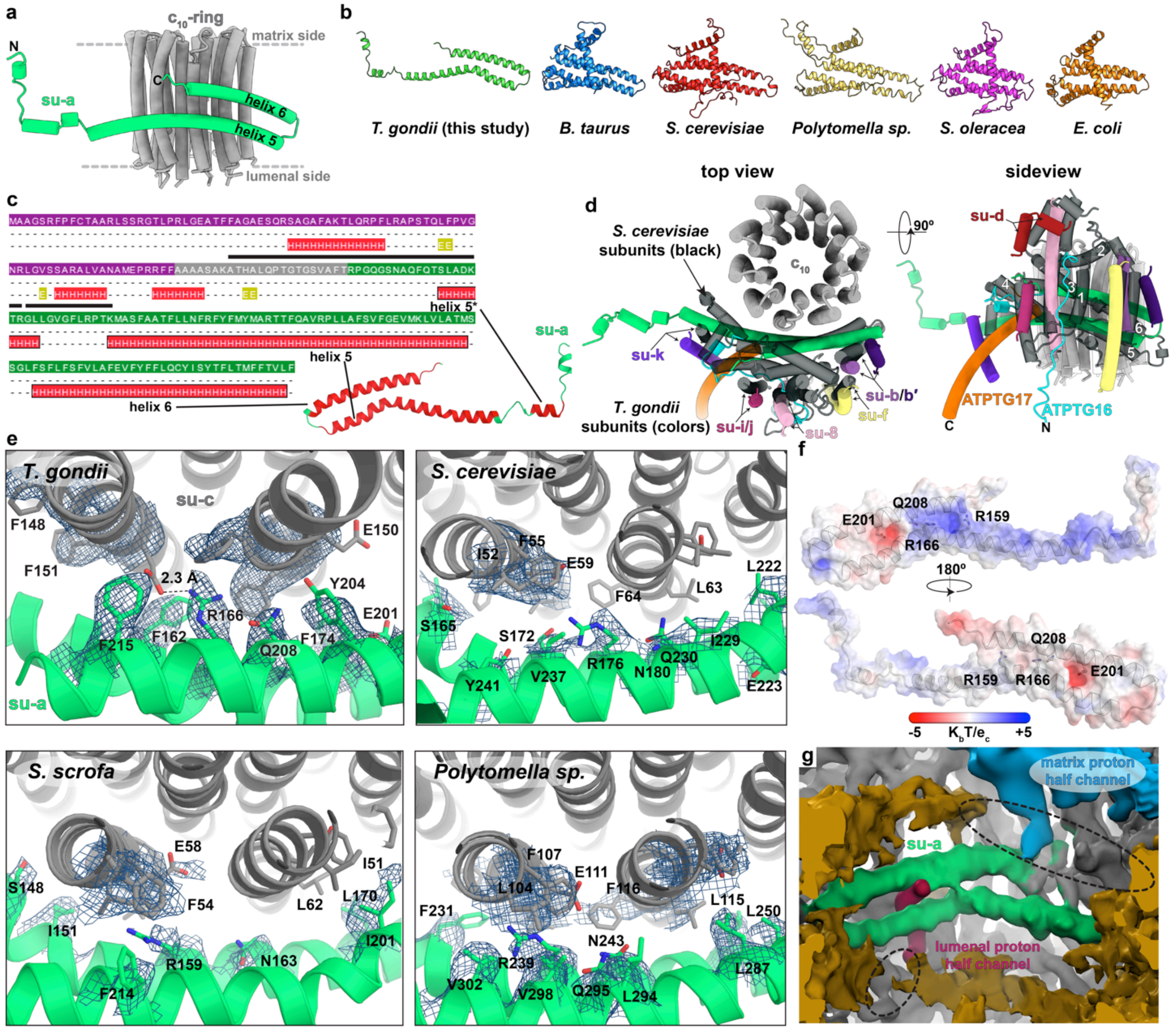

Supplementary Fig. 8 | Minimal subunit-*a* and rotor-stator interface.

a The structurally conserved H5$_a$ and H6$_a$ form interface with the c-ring. **b** Fold comparison of subunit-*a*. **c** Secondary structure prediction of *T. gondii* subunit-*a,* mt-signal sequence is purple. Modelled and unmodelled regions are green and grey, respectively. Black lines indicate the two peptides detected by previous mass spectrometry studies[4,5]. **d** Two views of the rotor-stator interface show structurally conserved subunits associated with the subunit-*a*. The superimposed *S. cerevisiae* structure[2] is transparent grey. **e** Comparison of subunit-*a/c* interfaces with other organisms shows that it contains fewer aromatic residues surrounding the central arginine/glutamate pair. **f** Electrostatic surface potential map of subunit-*a* highlighting the acidic patch around residue Glu201$_a$ where protons exit the lumenal proton half-channel onto the c-ring. **g** Proton half-channels shown in red (lumenal) and blue (matrix), and compared to gaps (dotted black circles) in detergent density (dark gold) of the cryo-EM density map of the hexamer.

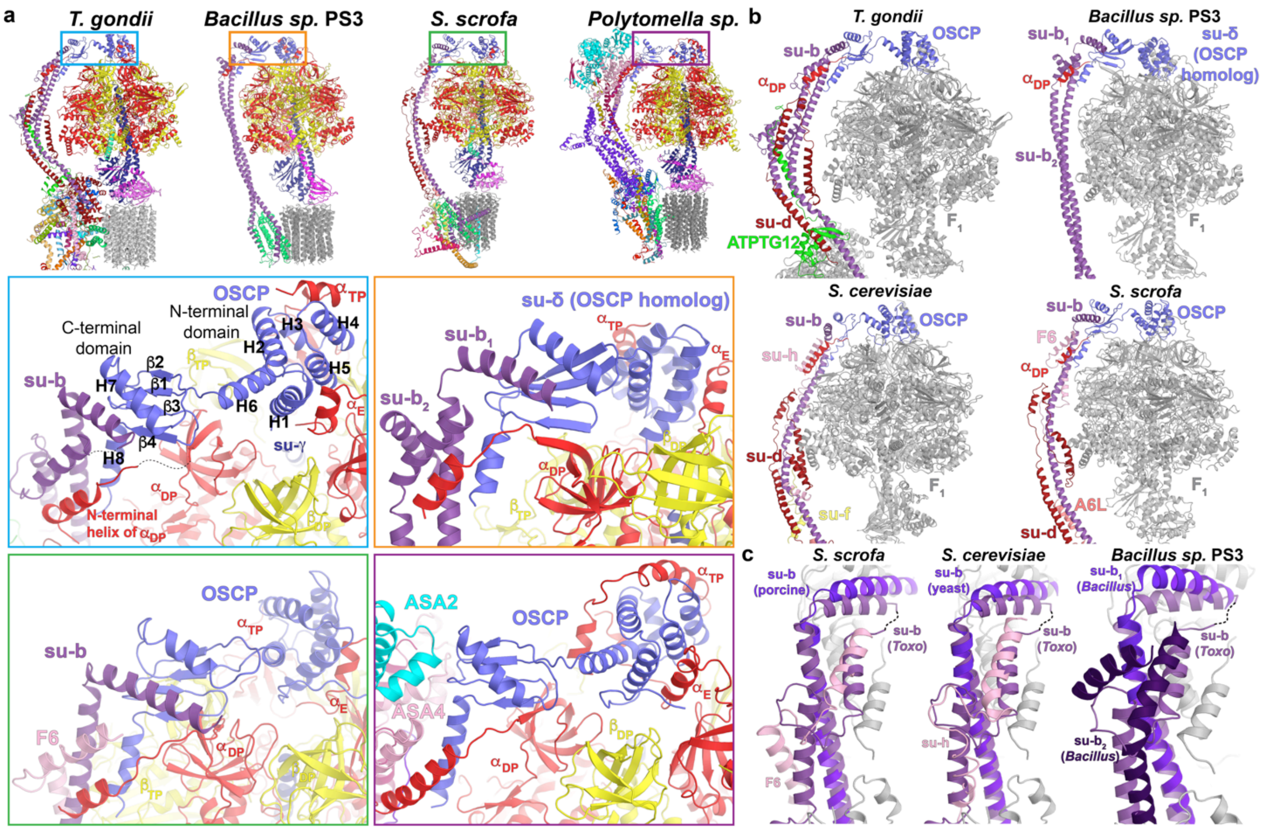

Supplementary Fig. 9 | Peripheral stalk and F_1 attachment.

a ATP synthase structures of *T. gondii* (this study), *Bacillus* PS3 (PDB 6N2Y) [http://doi.org/10.2210/pdb6N2Y/pdb], *S. scrofa* (PDB 6J5I) [http://doi.org/10.2210/pdb6J5I/pdb] and *Polytomella sp.* (PDB 6RD9) [http://doi.org/10.2210/pdb6RD9/pdb] with color-coded insets showing close-up views of the OSCP region. In the parasite, bacterial and porcine complexes, attachment of OSCP to the peripheral stalk occurs via a structurally conserved interaction to subunit-*b*, which is absent in *Polytomella.* **b** View of the F_1 and peripheral stalk of ATP synthases from *T. gondii*, *Bacillus* PS3 (PDB 6N2Y), *S. cerevisiae* (PDB 6CP6) [http://doi.org/10.2210/pdb6CP6/pdb] and *S. scrofa* (PDB 6J5I). **c** Superposition of the peripheral stalk shows that the C-terminal helix of *T. gondii* subunit-*b* superimposes closely with F6 and subunit-*h*, but not with the bacterial subunit-b_2, which has opposite topology.

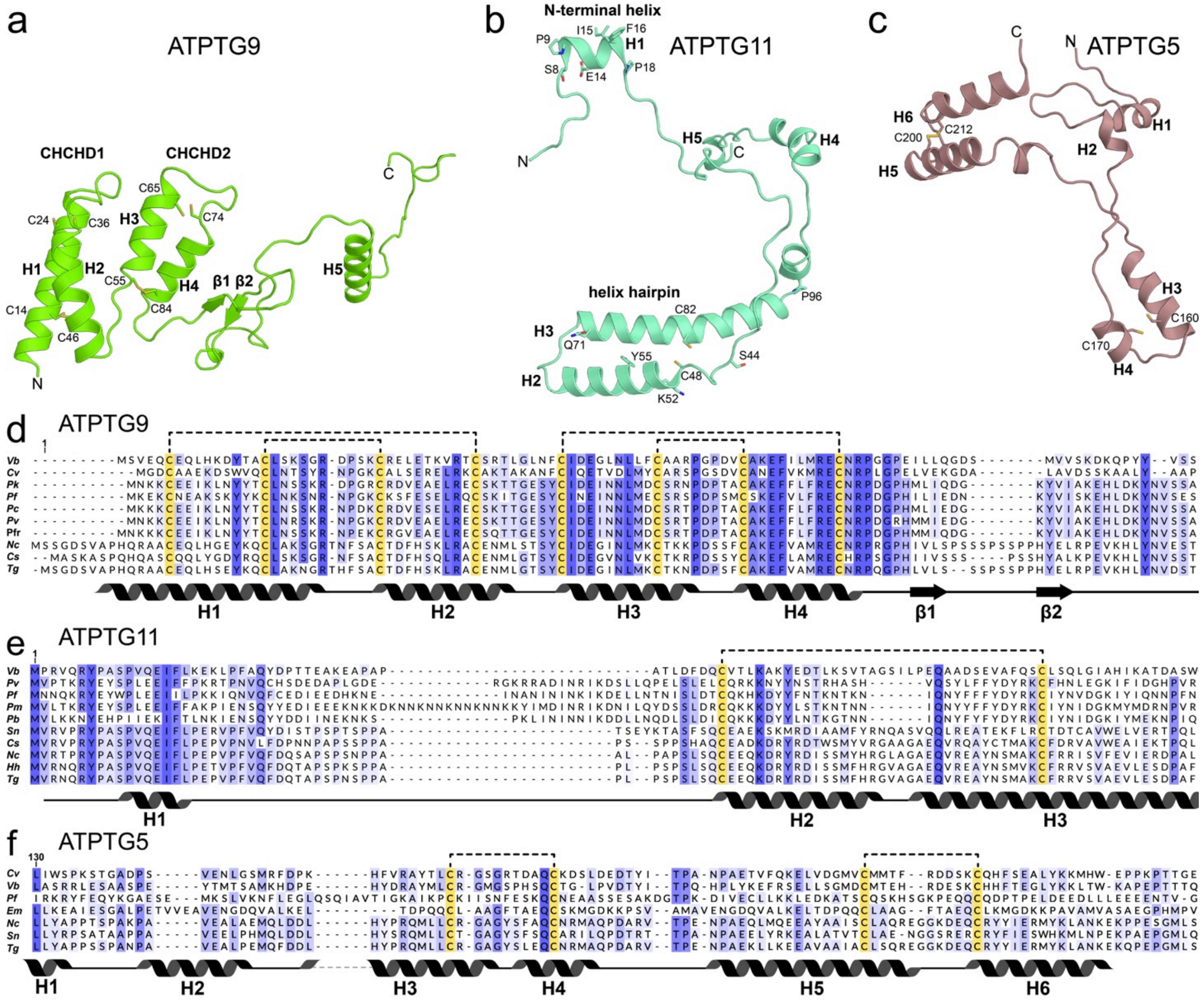

Supplementary Fig. 10 | Subunits of the hexamer interface are conserved in apicomplexan parasites.

a-c Structures of ATPTG9 (a) ATPTG11 (b) and ATPTG5 (c) with conserved residues indicated. ATPTG9 contains two CHCHD domains with a cysteine pair each. ATPTG11 contains a central cysteine pair in the helix hairpin mediating hexamer contacts. ATPTG5 contains two helix hairpins with one cysteine pair each. **d-f** Sequence alignments of ATPTG9 (d) ATPTG11 (e) and ATPTG5 (f) to protein sequences from different Apicomplexa, with conserved cysteines highlighted in yellow. Apicomplexan species: *Pk, Plasmodium knowlesi*; *Pf, Plasmodium falciparum*; *Pc*, *Plasmodium cynomolgi*; *Pv*, *Plasmodium vivax*; *Pfr*, *Plasmodium fragile*; *Pm*, *Plasmodium malariae*; *Pb*, *Plasmodium berghei*; *Nc*, *Neospora caninum; Sn, Sarcocystis neurona*; *Cs*, *Cystoisospora suis*; *Hh*, *Hammondia hammondi*; *Tg, Toxoplasma gondii*; and the non-parasitic alveolates: *Vb*, *Vitrella brassicaformis*; *Cv*, *Chromera velia.* Numbering and secondary structure annotations refer to *Toxoplasma gondii*.

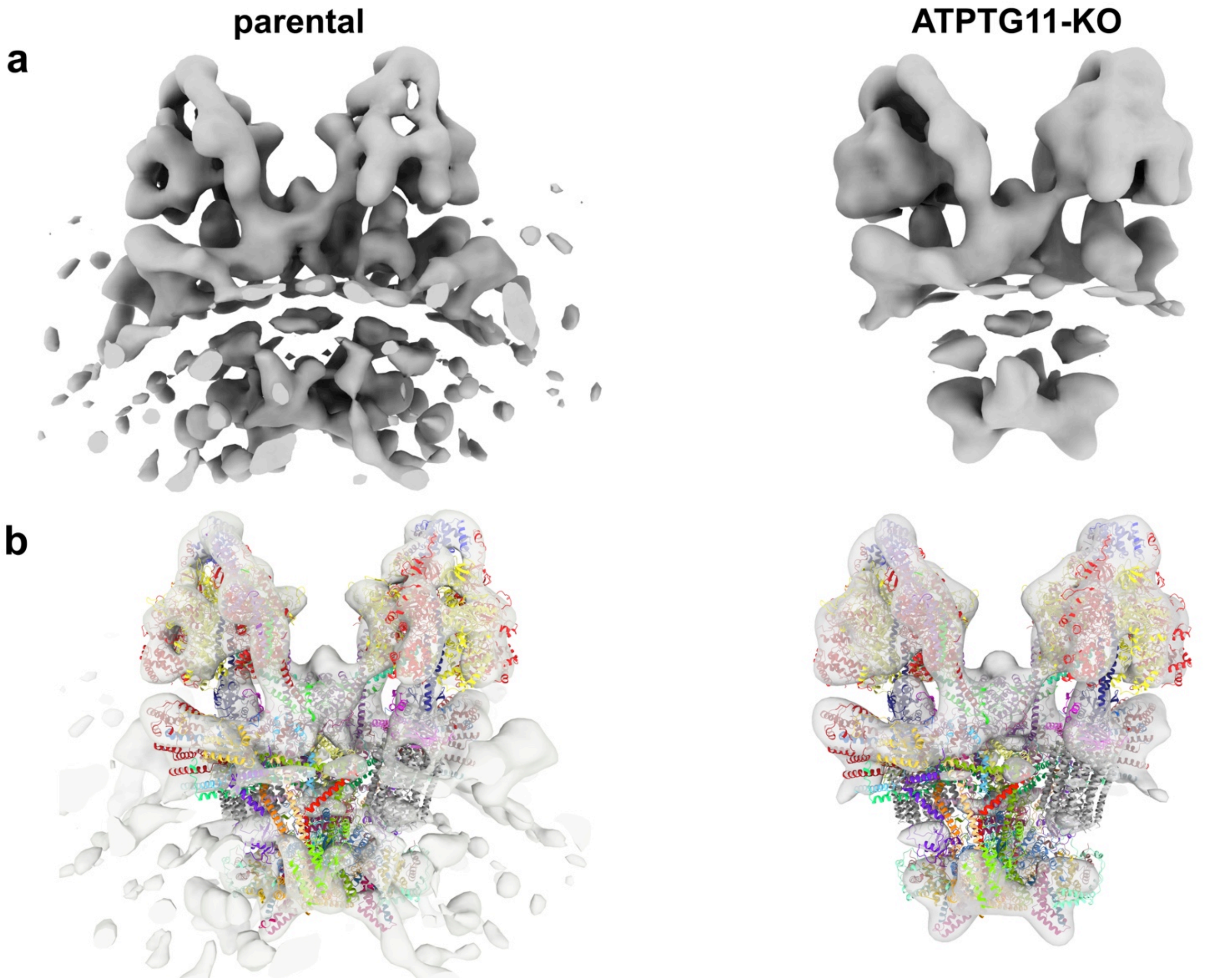

Supplementary Fig 11 | Subtomogram averages of *T. gondii* ATP synthase from parental and ATPTG11-KO mitochondrial membranes.

a. Subtomogram average maps of the dimers from parental (left), and ATPTG11-KO (right) membranes. **b** Subtomogram averages (transparent) with the atomic model fitted.

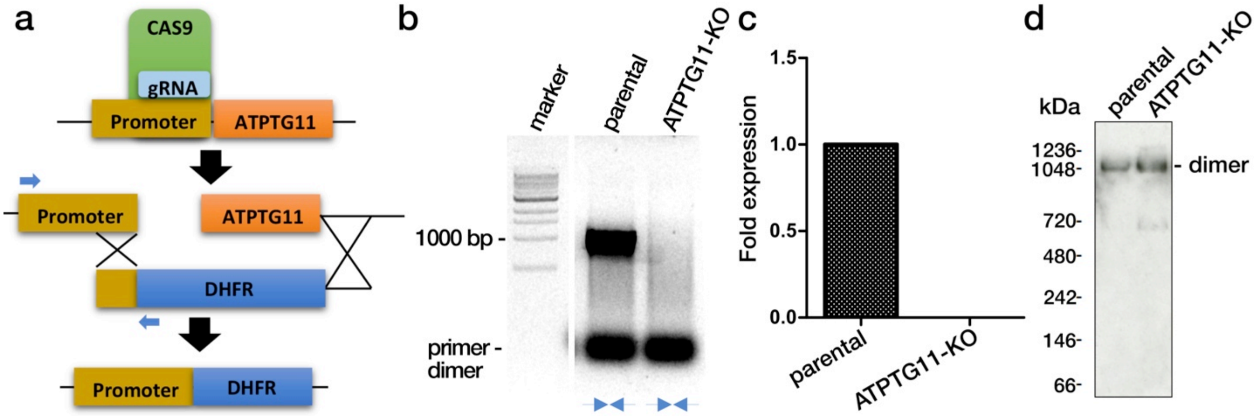

Supplementary Fig. 12 | ATP synthase dimers are formed in ATPTG11-KO.

a Schematic of genetic manipulation resulting in ATPTG11 knockout. Primers used for confirmation of insertion (generating a 1000-bp product only from knockout) shown as blue arrows. **b** Confirmation of the genetic manipulation of ATPTG11 locus via PCR. **c** Confirmation of absence of ATPTG11 mRNA in KO-strain via qRT-PCR. **d** Detection of ATP synthase dimers via native gel electrophoresis and western blotting using anti-ATP-β antibody.

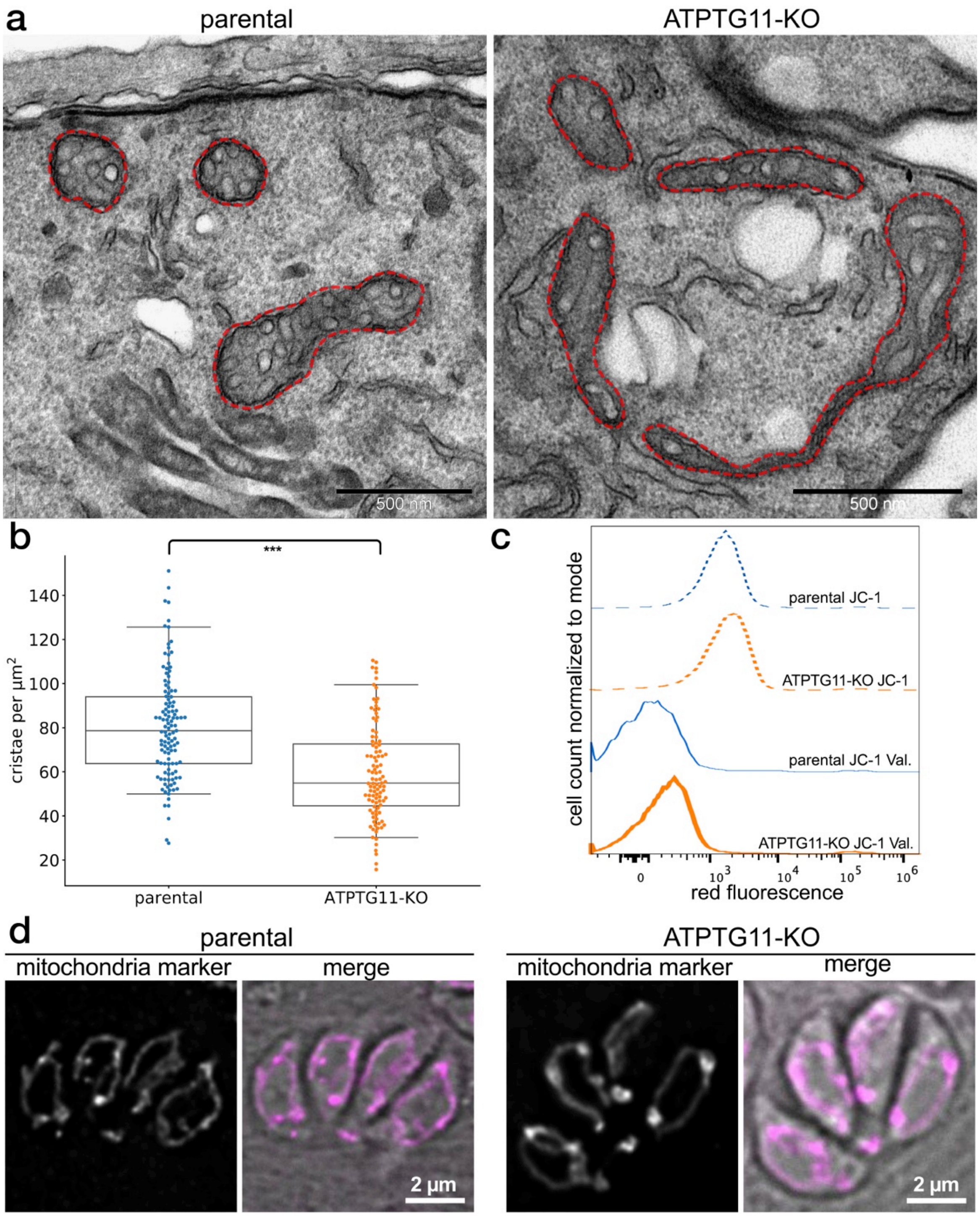

Supplementary Fig. 13 | Characterisation of the ATPTG11-KO line.

a Transmission electron microscopy of thin sections of *T. gondii* with mitochondria outlined (red dashed lines), showing cristae morphology. **b** Analysis of the number of cristae cross-sections per mitochondrial surface area for the parental and ATPTG11-KO strain. Box plot bounds range form first to third quartile with centre line indicating median; whiskers indicate 5/95th percentiles. ***$p=2.24 \times 10^{-10}$ by two-tailed Mann-Whitney test, n=118/104 mitochondria examined in parental and ATPTG11-KO, respectively. **c** Fluorescent flow cytometry analysis of *T. gondii* stained with the dye JC-1 indicated that both lines possess a mitochondrial membrane potential (parental blue, ATPTG11-KO orange; dotted), which is sensitive to the ionophor valinomycin (parental blue, ATPTG11-KO orange line). **d** Immunofluorescence light microscopy of the mitochondrial marker TgMys showing the lasso-shaped ultrastructure in both lines.

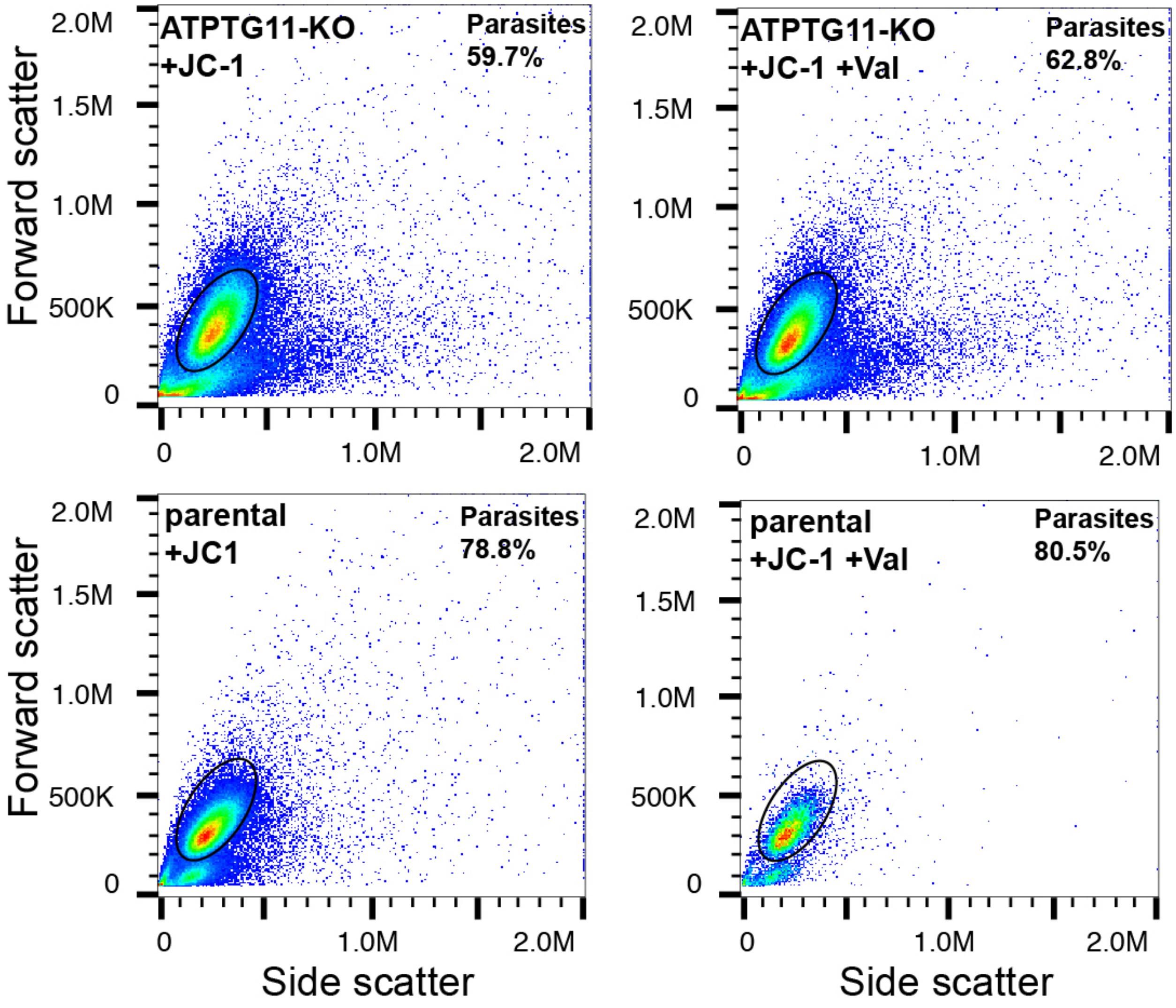

Supplementary Fig. 14 | Gating strategy for fluorescent flow cytometry experiments.
Each graph shows the forward scatter and side scatter plots of the entire population captured for each of the experiments shown in Supplementary Fig. 13C analysis. Oval gates were generated manually using FlowJo software (FlowJo LLC) to gate on the parasite population and separate it from host cell debris. The gated parasite populations (% from total event shown on the top left of each graph) were subsequently analysed for red fluorescence in the graphs shown in Supplementary Fig. 13C. The graphs for ATPTG11-KO+JC-1, parental+JC-1, ATPTG11-KO+JC-1+valinomyin and parental+JC-1+valinomycin represent n=5/8/2/8 biological replicates, respectively.

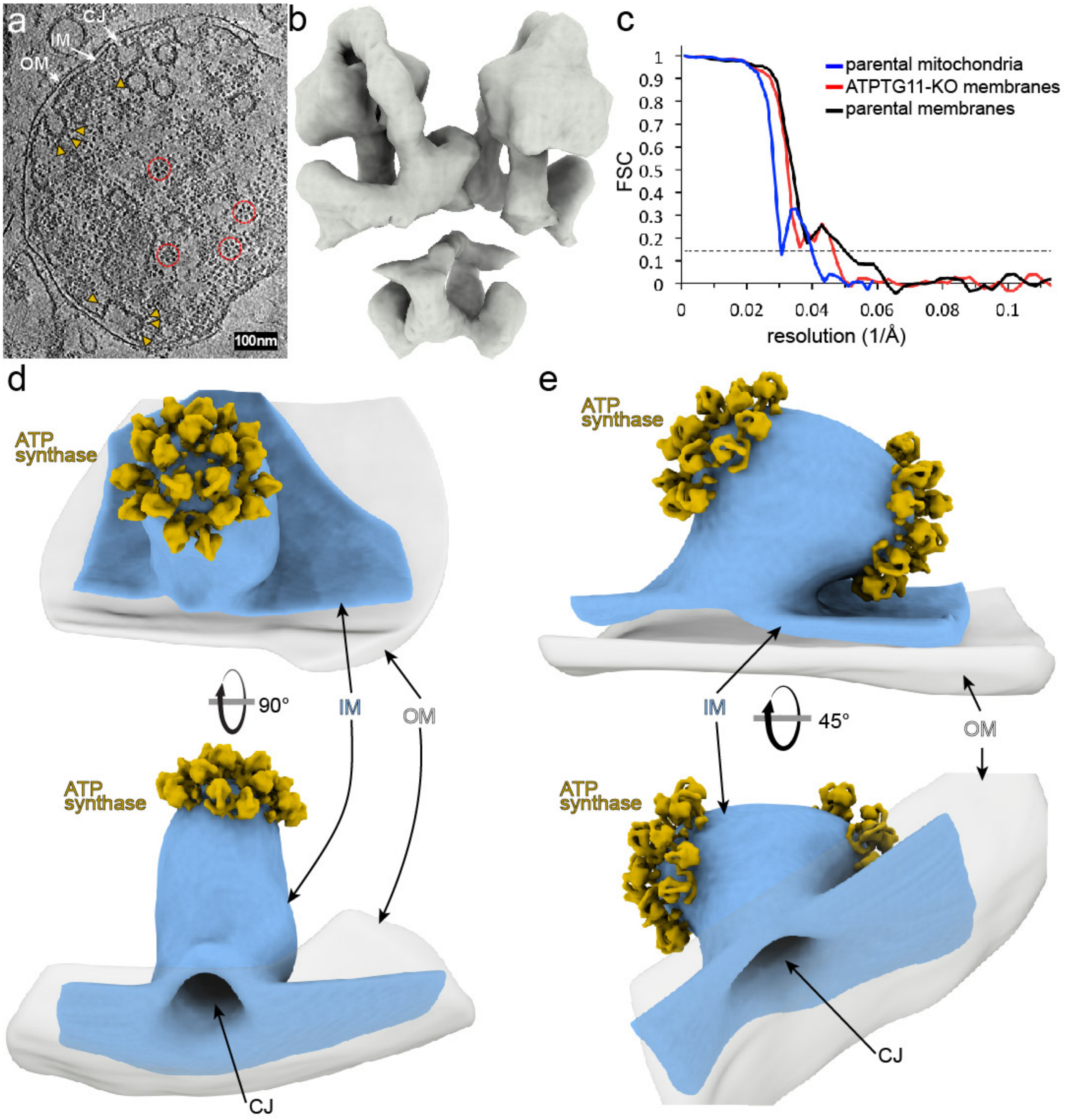

Supplementary Fig. 15 | Electron cryo-tomography of *T. gondii* mitochondria shows formation of pentagonal pyramid arrays *in organello*.

a Tomographic slice through a *T. gondii* mitochondrion, ATP synthase (yellow arrowheads) decorate the apical region of the cristae membranes. Top views of ATP synthase hexamers are highlighted with red circles (IM: inner membrane; OM: outer membrane; CJ: crista junction). **b** Subtomogram average of the ATP synthase dimer. **c** Fourier shell correlation (FSC) of the subtomogram averages; dashed line is 0.143-cutoff. **d,e** Subtomogram averages and segmented cristae (blue) showing ATP synthase pentagonal pyramid arrays (yellow) on the apices of the inner membrane.

Supplementary Table 1. Cryo-EM data collection, refinement and validation statistics.

	F_o dimer	OSCP/F_1-c-ring	Peripheral stalk	Rotor	F_1F_o dimer	F_1F_o hexamer
**Data collection**						
**Microscope**	Titan Krios					Titan Krios
**Voltage (kV)**	300					300
**Camera**	K2 Summit					K2 Summit
**Magnification**	165,000					130,000
**Exposure (e^-/Å^2)**	41.5					32
**Defocus range (µm)**	1.0-3.8					1.2-4.0
**Pixel size (Å)**	0.85					1.05
**Movies collected**	4860					7604
**Frames per movie**	20					20
**Data processing**						
**Initial particles**			275,030			26,542
**Final no. particles**	101,505	203,010	203,010	203,010	101,505	4,532
**Symmetry**	C_2	C_1	C_1	C_1	C_2	C_3
**Resolution (Å)***	2.8	3.1	3.5	3.5	2.9	4.8
**Sharpening B factor**	-75	-98	-112	-132	-79	-131
**EMDB ID**	**10520**	**10521**	**10522**	**10523**	**10524**	**10525**
**Model refinement statistics**						
**CC (map/model)**	0.85	0.85	0.77	0.83	0.66	0.45
**Resolution (map/model)**	2.70	3.05	3.93	3.57	3.0	9.0
**No. of atoms (no H)**	121,096	65,413	9,606	18,084	266,864	776,996
**No. of residues**	7,122	4,264	605	1,204	16,596	49,776
**No. of lipids**	44	-	-	-	44	-
**No. of ATP/ADP**	-	3/2	-	-	6/4	-
**No. of Mg ions**	-	5	-	-	10	-
**B-factor (Å^2)**	--					
**- protein**	41.24	50.62	102.30	56.53	48.38	49.64
**- lipids**	37.08	-	-	-	38.36	-
**- ATP/ADP**	-	31.68	-	-	38.36	-
**Rotamer outliers (%)**	0.69	0.29	0.58	0.00	0.25	0.01
**Ramachandran (%)**	--					
**- outliers**	0.00	0.00	0.00	0.00	0.02	0.04
**- allowed**	2.85	4.55	9.98	8.69	3.73	4.46
**- favored**	97.15	95.45	90.02	91.31	96.25	95.51
**Clash score**	1.30	2.61	3.54	2.43	1.87	2.00
**MolProbity score**	1.01	1.36	1.69	1.54	1.20	1.28
**RMSD**	--					
**- bonds (Å)**	0.004	0.006	0.004	0.006	0.004	0.002
**- angles (°)**	0.554	0.588	0.594	0.600	0.576	0.567
**EMRinger score**	4.49	3.59	1.38	2.54	3.15	0.54
**PDB ID**	**6TMG**	**6TMH**	**6TMI**	**6TMJ**	**6TMK**	**6TML**

Supplementary Table 2: *T. gondii* ATP synthase subunit composition

Subunit	Previous nomenclature [4]	Previous nomenclature [5]	Gene entry	Uniprot ID	Predicted length (aa)	Residues built	M_w modeled (Da)	Comments
**<u>F_1-subcomplex:</u>**								
α	α	α	TGGT1_204400	S7UU80	577	53-564	55910	
β	β	β	TGGT1_261950	A0A125YYY4	560	79-557	51771	attempts to isolate KO in *P. falciparum* were unsuccessful; KO in *P. berghei* is lethal in the ookinetes [6]
γ	γ	γ	TGGT1_231910	A0A125YUH0	314	42-313	30257	attempts to isolate KO in *P. falciparum* were unsuccessful [6]
δ	δ	δ	TGGT1_226000	A0A125YRE2	183	41-183	15256	
ε	ε	ε	TGGT1_314820	S7VV10	73	1-65	7686	
OSCP	OSCP	OSCP	TGGT1_284540	A0A125YKG0	252	73-252	20134	
**Inhibitor of F_1 (IF_1)**	putative IF1	-	TGGT1_215350	A0A125YJP2	145	57-96	4777	
**<u>F_o-subcomplex:</u>**								
a	putative a	ASAP-1	TGGT1_31036	A0A125YSJ0	224	108-224	13439	

			0					
b	ICAP2	ASAP-2	TGGT1_231410	A0A125YYF7	571	83-571	55742	
c	c	c	TGGT1_249720	A0A125YJV2	166	96-166	7170	
d	ICAP18	ASAP-3	TGME49_268830	S8F0X4	536	20-100, 107-288, 304-507	53597	
f	ICAP11	ASAP-10	TGGT1_215610	S8ESP4	111	2-111	12883	
i/j	-	ASAP-11	TGGT1_290030	A0A125YIW7	229	47-222	21225	
k	ICAP6	ASAP-6	TGGT1_260180	A0A125YTD8	157	9-157	17250	PB1/UBX domain
8	-	ASAP-15	TGGT1_208440	A0A125YPQ5	205	96-191	10871	
ATPTG1	-	-	TGGT1_246540	S7W9J5	398	32-153	14523	
ATPTG2	ICAP15	ASAP-8	TGGT1_282180	A0A125YV77	310	42-213, 229-310	29334	
ATPTG3	ICAP8	ASAP-7	TGGT1_218940	S8G8Q0	325	164-303	15773	
ATPTG4	-	ASAP-16	TGGT1_201800	A0A125YM53	267	71-102, 112-267	20426	thioredoxin-like fold
ATPTG5	-	ASAP-20	TGGT1_270360	S8F1E8	252	115-226	12542	
ATPTG6	unknown	ASAP-4	TGGT1_223040	S8FBB2	239	14-239	26191	
ATPTG7	-	-	TGGT1_290710	S7VXW3	236	146-235	10196	CHCHD
ATPTG8	CHCH domain	-	TGGT1_258060	S7W7F1	208	2-208	23203	CHCHD
ATPTG9	CHCH domain	ASAP-9	TGGT1_285510	S8F8M0	166	7-166	17807	CHCHD KO attempts unsuccessful
ATPTG10	-	ASAP-13	TGGT1_214930	A0A125YMA8	138	15-122	12292	
ATPTG11	-	ASAP-19	TGGT1_263990	A0A125YPS5	134	2-134	15275	KO line generated in this study

ATPTG12	-	ASAP-14	TGGT1_245450	A0A125YKF9	134	1-133	15251	
ATPTG13	-	ASAP-17	TGGT1_225730	A0A125YLN5	128	33-127	11271	
ATPTG14	-	ASAP-18	TGGT1_263080	A0A125YLI0	133	18-109	10281	
ATPTG15	ICAP9	ASAP-5	TGGT1_247410	A0A125YRP2	126	33-126	10642	
ATPTG16	-	-	TGGT1_211060	S7VTI0	106	2-105	10679	
ATPTG17	-	ASAP-12	TGGT1_310180	S8G3L1	92	2-83	9347	

ATPTG1, ATPTG7 and ATPTG16 were not detected in any of the previous studies, and built *de novo* through building peptide sequences in the density map of F_o and performing BLAST searches of the built sequences against the ToxoDB.

Supplementary Table 3: Primers used in this study

**Primer name**	**Sequence**
Forward primer to clone gRNA	aagttGATTGCGCACCATCTTGCACg
Reverse primer to clone gRNA	aaaacGTGCAAGATGGTGCGCAATCa
Forward primer to amplify DHFR	ccccacgccggttcgagttggcagtcgatttcttccatagcccgtgcaagaagcttcgc caggctgtaaatcc
Reverse primer to amplify DHFR	CTACAAGTGGTACTTCGTTAGAATGCTCGGCACTCT CCCATACTGCAGCTggatcgatcccccggtttgc
Integration testing primer 1	GCCGTTGTGCTGCG
Integration testing primer 2	GGCCGTTGTTGATGCC
Actin forward primer KO verification	GGGACGACATGGAGAAAATC
Actin forward primer KO verification	AGAAAGAACGGCCTGGATAG
ATPTG11-KO forward primer KO verification	CTCCACTCCCTTCTCCCTC
ATPTG11-KO reverse primer KO verification	ATGGTGAAGTTCCGAGCTTG
Catalase forward primer competition assay	TCCCCAACTGTTGTTACCGT
Catalase reverse primer competition assay	CAGCGTTTCCTGTTAATGCAG
ATPTG11-KO forward primer competition assay	ACCCGTCGACAATTTCGCTA
ATPTG11-KO reverse primer competition assay	CCTGAGGACACCTGCTTGAT
Parental ATPTG11 forward primer competition assay	GACAATTTCGCTAAATCCTCCC
Parental ATPTG11 reverse primer competition assay	CGAACTGGACAAACGGGAC

Primers used for the generation and verification of the ATPTG11-KO line and the growth competition assay. For the primers used to clone gRNA, gRNA nucleotides are shown in uppercase, nucleotides that create restriction site in lowercase.

Supplementary Information References

1 Murphy, B. J. *et al.* Rotary substates of mitochondrial ATP synthase reveal the basis of flexible F1-Fo coupling. *Science* **364**, doi:10.1126/science.aaw9128 (2019).

2 Guo, H., Bueler, S. A. & Rubinstein, J. L. Atomic model for the dimeric FO region of mitochondrial ATP synthase. *Science* **358**, 936-940, doi:10.1126/science.aao4815 (2017).

3 Gu, J. *et al.* Cryo-EM structure of the mammalian ATP synthase tetramer bound with inhibitory protein IF1. *Science* **364**, 1068-1075, doi:10.1126/science.aaw4852 (2019).

4 Huet, D., Rajendran, E., van Dooren, G. G. & Lourido, S. Identification of cryptic subunits from an apicomplexan ATP synthase. *eLife* **7**, doi:10.7554/eLife.38097 (2018).

5 Salunke, R., Mourier, T., Banerjee, M., Pain, A. & Shanmugam, D. Highly diverged novel subunit composition of apicomplexan F-type ATP synthase identified from *Toxoplasma gondii*. *PLoS Biol* **16**, e2006128, doi:10.1371/journal.pbio.2006128 (2018).

6 Balabaskaran Nina, P. *et al.* ATP synthase complex of Plasmodium falciparum: dimeric assembly in mitochondrial membranes and resistance to genetic disruption. *J Biol Chem* **286**, 41312-41322, doi:10.1074/jbc.M111.290973 (2011).
