# Supplementary material for: ATP synthase hexamer assemblies shape cristae of Toxoplasma mitochondria
Source: Nat Commun. 2021 Jan 5;12:120. doi: 10.1038/s41467-020-20381-z (PMC7785744; doi:10.1038/s41467-020-20381-z)
Supplement: Supplementary file 6 — Description of Additional Supplementary Files [file 41467_2020_20381_MOESM6_ESM.docx]

Description of additional supplementary files

Title: Supplementary Movie 1

Description: CryoEM-map of the *T.gondii* ATP synthase hexamer

Title: Supplementary Movie 2

Description: Electron cryo-tomography of *T. gondii* mitochondrial membranes reveals the macromolecular arrangement of ATP synthase into pentagonal pyramids

Title: Supplementary Movie 3

Description: Electron cryo-tomography of ATPTG11-KO mitochondrial membranes reveals altered macromolecular arrangement of ATP synthase dimers
